# From Primary Melanoma to Metastatic Evolution: AI-Powered Pathology Integrated with Functional Analysis and Clinical Metadata Improving Treatment Prediction

**DOI:** 10.3390/cancers18121951

**Published:** 2026-06-16

**Authors:** Lívia Fülöp, Leticia Szadai, Balazs Szigeti, Lukas Christersson, Henriett Oskolas, Peter Horvatovich, Diana Lashidua Fernandez-Coto, Johan Malm, Elisabet Wieslander, Bo Baldetorp, Sergio Encarnación-Guevara, Attila Marcell Szasz, Istvan Balazs Nemeth, David Fenyö, Jeovanis Gil, György Marko-Varga

**Affiliations:** 1 National Kerenyi Institute of Pulmonology, 1122 Budapest, Hungary; fulop.livia@koranyi.hu (L.F.); szigeti.balazs@semmelweis.hu (B.S.); 2 Section for Clinical Chemistry, Department of Translational Medicine, Lund University, 22184 Lund, Sweden; leticia.szadai@med.lu.se (L.S.); lukas.christersson@med.lu.se (L.C.); henriett.kovacs-oskolas@med.lu.se (H.O.); dianafer@ccg.unam.mx (D.L.F.-C.); johan.malm@med.lu.se (J.M.); elisabet.wieslander@med.lu.se (E.W.); jeovanis.gil_valdes@med.lu.se (J.G.); 3 Department of Dermatology and Allergology, Albert Szent-Györgyi Medical School, University of Szeged, 6720 Szeged, Hungary; nemeth.istvan.balazs@med.u-szeged.hu; 4 Department of Analytical Biochemistry, Faculty of Science and Engineering, University of Groningen, 9713 AV Groningen, The Netherlands; p.l.horvatovich@rug.nl; 5 Center for Genomic Sciences, National Autonomous University of Mexico, Cuernavaca 62210, Mexico; encarnac@ccg.unam.mx; 6 Division of Oncology, Department of Clinical Sciences Lund, Lund University, 22184 Lund, Sweden; bo.baldetorp@med.lu.se; 7 Department of Internal Medicine and Oncology, Semmelweis University, 1083 Budapest, Hungary; szasz.attila.marcell@semmelweis.hu; 8 Department of Biochemistry and Molecular Pharmacology, NYU Grossman School of Medicine, New York, NY 10016, USA; david.fenyo@nyulangone.org; 9 Institute for Systems Genetics, NYU Grossman School of Medicine, New York, NY 10016, USA; 10 Department of Biotechnology, College of Life Science and Biotechnology, Yonsei University, Seoul 03722, Republic of Korea; 11 1st Department of Surgery, Tokyo Medical University, Tokyo 160-8582, Japan

**Keywords:** AI-assisted computational pathology, melanoma, tumor, metastasis, imaging, proteomics

## Abstract

A critical gap in current efficiency in melanoma patient treatment is the lack of a fully integrated, functional understanding of tumor evolution over time. Recent advances have fundamentally reshaped our understanding of melanoma biology, while increasing clinical complexity has highlighted the need for more comprehensive and biologically informed clinical decision-support frameworks. We propose the implementation of a multimodal disease profiling framework as a core clinical decision-support asset, enhancing treatment optimization across the full disease course in melanoma patients. By integrating proteogenomics, AI-driven digital image analysis, and structured longitudinal clinical metadata, multimodal disease profiling could provide a comprehensive and dynamically evolving view of each patient’s disease. Proteogenomics reveals tumor signaling activity, protein complex dynamics, and emerging therapeutic vulnerabilities that may drive progression and resistance. In parallel, AI-enabled digital pathology analysis characterizes tumor morphology, clonal heterogeneity, and immune context, capturing spatial and functional changes associated with metastatic transition. When combined with longitudinal clinical data, these layers enable patient-specific models tracking tumor evolution, metastasis, and treatment exposure. Leveraging one of the largest melanoma biobank and database resources at the European Cancer Moonshot Center in Lund, our strategy directly addresses the recurrent transition from primary tumors to metastatic disease. This strategy positions multimodal disease profiling as a critical enabler of precision melanoma care by providing biologically grounded, evidence-based decision support, facilitating rapid and structured case assessment through multimodal insights, enabling prediction of treatment response, resistance, and disease trajectory, and supporting adaptive, evidence-informed therapeutic decision-making.

## Simple Summary

Melanoma care is becoming increasingly complex, especially as more patients live longer with recurrent or metastatic disease. Although current multidisciplinary tumor boards combine clinical, pathological, and radiological information, important aspects of tumor biology are still not fully captured by routine assessment alone. This manuscript highlights how integrating AI-based image analysis, proteogenomics, and longitudinal clinical data can provide a more complete picture of disease behavior over time. This combined approach helps identify tumor heterogeneity, microenvironmental changes, immune interactions, and functional molecular programs linked to progression, recurrence, and treatment resistance. It also supports more structured case presentation and more precise interpretation of patient-specific disease evolution. By using standardized workflows and large-scale melanoma resources, this framework may improve risk stratification, treatment selection, and ongoing monitoring. Overall, this work highlights that melanoma management should move beyond static diagnostics toward an adaptive, biologically informed decision-support system for personalized oncology.

## 1. Introduction

Melanoma incidence and prevalence continue to rise globally, with an expected 510,000 cases per year, and nearly 100,000 deaths annually by 2040 [1]. The number of new invasive melanomas has steadily risen, growing by 32% over the last decade. Projections indicate > 330,000 new cases annually in recent years [2].

At least one in five Americans will develop skin cancer by age of 70 [3], and Scandinavia reports among the highest melanoma incidence rates worldwide [4]. This underscores the urgent need for more effective strategies in modern healthcare to improve patient treatment and outcomes.

Today, clinical decision-making in oncology, globally, is increasingly conducted within a specialized medical multidisciplinary team, where treatment strategies are determined. In the frontline of this process is the treating oncologist, whose clinical management decisions are informed by comprehensive diagnostic input, with the pathological characterization of the tumor playing a aparticularly central role. Here, pathology reports provide critical insights into the disease presentation, including the tumor type, molecular features, and biological behavior, which form the opportunity for personalized treatment planning [5].

Modern tumor boards evolve from core medical specialties, primarily discipline-based decision forums, into novel science-driven, data-integrative platforms, where clinical expertise is augmented by advanced computational and molecular analytics [6]. Thus, core medical specialists, including radiologists and nuclear medicine physicians responsible for imaging and staging, surgeons assessing interventional strategies, and diagnostic and molecular experts interpreting biomarker and genomic profiles, all remain central to clinical decision-making [7]. Clinical pharmacologists and oncology pharmacists further guide therapeutic decisions, dosing strategies, and clinical trial inclusion, particularly with the emergence of targeted and cellular therapies. Palliative care specialists and genetic counselors contribute essential perspectives on patient quality of life (e.g., the EORTC QLG Core Questionnaire) [8] and hereditary risk assessment.

The rapid advancement of AI-driven technologies and precision oncology frameworks contributes to the fundamental transformation of this landscape [7]. Tumor boards incorporatedigital image analysis of pathology supported by AI imaging as key pillars, enabling high-resolution, quantitative characterization of tumor architecture, spatial heterogeneity, and microenvironmental interactions directly from histopathological data. In parallel, functional deep-mining approaches, including quantitative proteomics and proteogenomics, provide mechanistic insights into tumor signaling, protein complex dynamics, and therapy response at an unprecedented depth [9].

Emerging evidence suggests that this transformation is further supported by the integration of bioinformaticians and molecular data scientists, who enable multimodal data fusion across imaging, omics, and clinical metadata, converting complex datasets into actionable insights [10]. Together, these advances shift tumor board decision-making toward a quantitative, evidence-based, and mechanistically grounded paradigm, where “novel science” is not only supportive but becomes the central driver of patient-specific treatment strategies and predictive oncology [7]. The multimodal integration of clinical data, AI-based histopathology, and proteomics remains an emerging area with very limited published literature [11,12,13]. Our work represents one of the first systematic frameworks combining all three modalities in melanoma.

## 2. Clinical Presentation, Disease Evolution, and the Need for Integrated Decision-Making

Melanoma progression is increasingly recognized as a dynamic and evolving process characterized by complex interactions between tumor biology, immune response, treatment pressure, and disease evolution. While only a minority of patients present with stage IV disease at diagnosis, a substantial proportion of metastatic melanoma arises through recurrence from high-risk stage II–III disease. Historically, metastatic progression was associated with poor prognosis and median survival measured in months. Globally, prior to 2015, approximately 30–50% of patients developed distant metastases over time. However, the therapeutic landscape has changed dramatically over the past decade following the introduction of immune checkpoint inhibitors and targeted therapies (Table 1).

Between 2015 and 2025, a major epidemiological and clinical shift occurred, characterized by a substantial increase in the prevalence of metastatic melanoma, with the number of patients living with metastatic disease rising by approximately two- to threefold in some regions [14]. This transformation was driven by the introduction of immune checkpoint and targeted therapies, notably ipilimumab and vemurafenib in 2011, followed by the early adoption of PD-1 inhibitors shortly thereafter. As a result, in one recent case, we processed 41 metastatic tumor tissues from the same patient [5], reaching a conclusive treatment proposal that was functionally verified. One major challenge that was not obvious or expected was the ability to retrieve the tumor samples together with clinical data from five respective medical centers in multiple countries. Increasing clinical complexity has highlighted the need for game-changing breakthroughs.

In daily patient care across national cancer centers and hospitals with dedicated oncology departments, we observe a significant shift in metastatic melanoma. Prolonged patient survival enabled by immunotherapy and targeted treatments has transformed the disease into a dynamic, long-term condition. This evolution introduces new clinical challenges that demand updated directives, resources, and care strategies [14,15,16,17].

This shift has created new challenges for oncology care. Effective management increasingly requires characterization of tumor heterogeneity, therapy-induced biological adaptation, and mechanisms of resistance that may emerge during disease progression. Conventional clinicopathological variables remain essential but are often insufficient to capture the full biological complexity underlying treatment response and disease trajectory. Consequently, there is growing interest in multimodal approaches that integrate clinical metadata, digital pathology, radiological imaging, molecular profiling, and functional biological measurements into a unified analytical framework [5,18,19].

**Figure 1:**
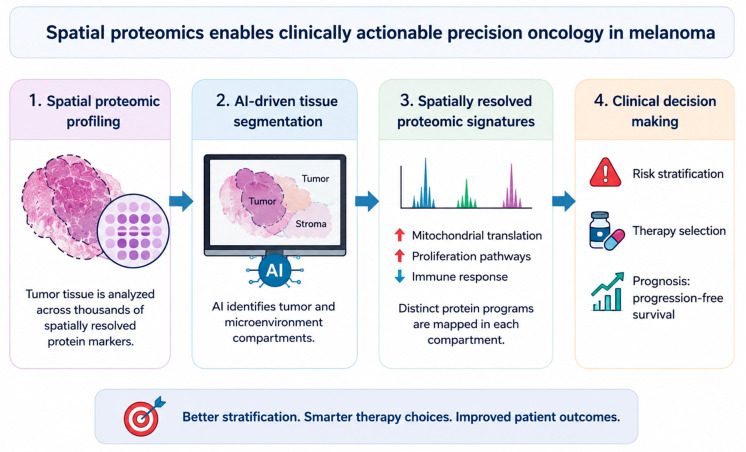
Schematic illustration of the process workflow at the European Cancer Moonshot Center. It shows how spatially resolved proteomic profiling combined with AI-assisted tissue analysis can reveal distinct biological signatures associated with proliferation, metabolic activity, immune regulation, and treatment response, supporting personalized therapeutic strategies [18]. Created using AI-assisted tools on BioRender.com [20].

Recent advances in AI-assisted digital pathology have enabled quantitative assessment of tissue architecture and microenvironmental features at single-cell resolution, while proteomic and other molecular approaches provide complementary insights into tumor function and biological activity. Routine clinical assessment may fail to reflect morphology and topography. The integration of these data streams creates opportunities for more precise patient stratification, improved prediction of therapeutic response, and adaptive clinical decision-making (Figure 1).

The field is entering a transformative era in which the growing complexity of melanoma management has underscored the importance of multidisciplinary tumor boards (MDTs), where specialists integrate clinical, pathological, radiological, and molecular information to guide optimal treatment decisions. Contemporary MDTs increasingly incorporate digital pathology, molecular diagnostics, and AI-assisted analytical tools as complementary sources of evidence. Clinical decision-making remains anchored in established frameworks, such as AJCC staging, ECOG performance status assessment, organ function evaluation, and international treatment guidelines, including those from ESMO [21] and NCCN [22]. However, the growing volume and complexity of available patient data highlight the need for integrated computational support systems capable of synthesizing heterogeneous information into clinically actionable outputs.

The fragmentation of healthcare data remains a major challenge across pathology systems, radiology archives, laboratory information management systems, and electronic health records. Although large-scale infrastructures such as the Swedish National Patient Register [23], the NCI SEER program [24], Flatiron Health [25], and the emerging European Health Data Space [26] demonstrate the potential of longitudinal data integration, interoperability and standardization remain significant barriers. Future precision oncology frameworks will likely depend on harmonized data models, structured digital reporting, AI-assisted extraction tools, and continuously updated patient repositories capable of integrating clinical, imaging, pathological, and molecular information. Such systems may ultimately support more efficient MDT workflows, facilitate evidence-based treatment selection, and accelerate the implementation of truly personalized oncology care.

## 3. The Illustrative Institutional Pipeline

### 3.1. Current Status of Multimodal AI Approaches and AI-Based Tumor Boards

Recent initiatives in artificial intelligence have accelerated the development of multimodal oncology frameworks that integrate heterogeneous data sources, including clinical records, digital pathology, radiology, genomics, transcriptomics, and proteomics. Emerging advances have fundamentally reshaped our understanding of cancer biology, and rather than relying on a single modality, these approaches aim to capture the complex biological and clinical heterogeneity of cancer through data fusion strategies that improve patient stratification, prognostic assessment, and treatment prediction [27,28]. In melanoma, independent groups have demonstrated the value of multimodal integration. For example, Andrew et al. developed MelanoMAP, a multimodal AI framework integrating tumor microenvironment-derived digital biomarkers with clinicopathological variables from more than 3500 histology slides, achieving a substantial improvement in metastasis prediction compared with conventional AJCC staging [29]. Similarly, recent reviews of digital pathology and AI in melanoma have highlighted the rapid evolution of whole-slide-image-based classification, spatial modeling, tumor-infiltrating lymphocyte quantification, and molecular prediction approaches that increasingly combine histopathologic and clinical information [12]. Beyond melanoma, multimodal deep learning has emerged as a major research direction in oncology, with AI systems integrating imaging, molecular profiling, and longitudinal clinical data across multiple cancer types [13]. Furthermore, the growing concept of oncological digital twins has positioned AI as a key technology for integrating multi-omics data, ranging from genomics and transcriptomics to proteomics, with clinical and imaging information to support precision medicine [30]. The clinical implementation of molecular tumor boards and precision oncology decision-support systems [31] has expanded substantially in recent years, with academic cancer centers (e.g., Cedars-Sinai Center [32,33], CA, USA; Dana-Farber Cancer Institute [34], MA, USA; University Hospital Zurich [35], Switzerland; Karolinska University Hospital, Sweden [31]) increasingly incorporating genomic and multimodal data into treatment planning through dedicated decision-support platforms and multidisciplinary molecular tumor boards. Based on this background, the novelty of the present perspective lies not in proposing AI-assisted tumor boards per se, but in linking compartment-resolved spatial proteomics, AI-driven digital pathology, and longitudinal clinical metadata within a structured pipeline. With continued advances in the field, AI-driven digital pathology utilizing foundation models, among others, is expected to become a cornerstone of future pathology reporting and clinical decision-making in oncology [36,37,38]. Importantly, we acknowledge that this framework represents a future-oriented translational vision requiring prospective validation rather than a clinically deployable system at present. Such approaches may also contribute to the principles of green oncology by improving patient stratification, reducing redundant testing, and supporting more efficient utilization of healthcare resources [7].

### 3.2. Establishing a Final Decision-Making Output Framework with Integrated Electronic Patient Progress Tracking

**Figure 2:**
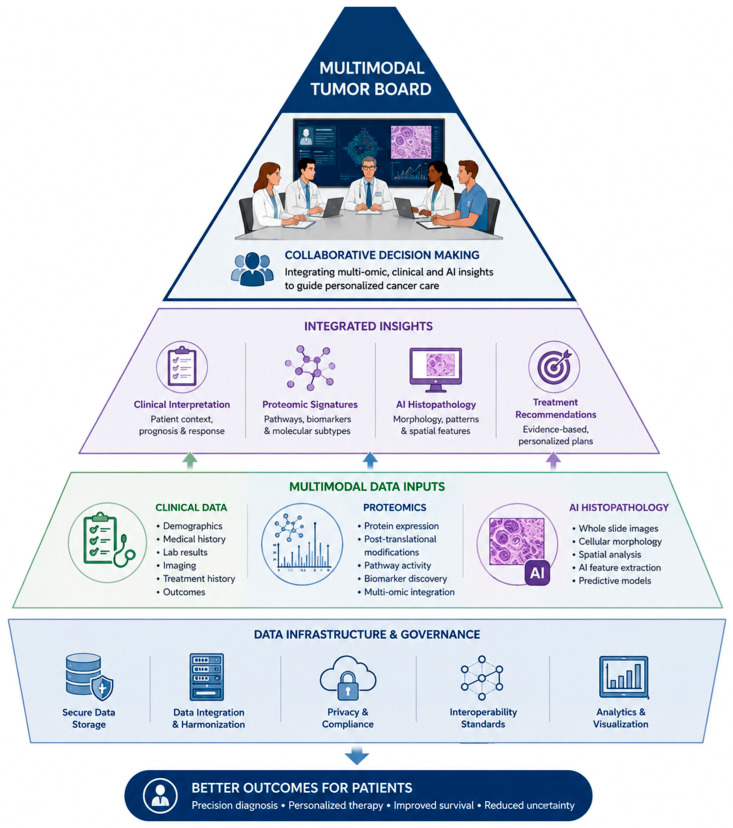
The Illustrative Institutional Pipeline pyramid. This schematic illustrates an integrated, repository-driven tumor board pipeline that unifies digital computational pathology, AI-based imaging, proteomic profiling, and comprehensive clinical metadata, including longitudinal patient history.

Our Illustrative Institutional Pipeline represents a next-generation, data-centric framework designed to integrate digital computational pathology, AI-based imaging, proteomic profiling, radiological assessment, molecular characterization, and longitudinal clinical metadata within a unified analytical environment. By continuously capturing, structuring, and analyzing multimodal patient data, the platform enables the real-time synthesis of disease evolution, treatment response, and predictive insights (Figure 2).

Such approaches are increasingly recognized as a key direction in precision oncology, where artificial intelligence serves as a bridge between heterogeneous data modalities, including clinical records, histopathology, imaging, genomics, transcriptomics, and proteomics, to support more comprehensive patient stratification and therapeutic decision-making [13,30].

The framework is centered around the creation of a continuously updated electronic patient overview that integrates longitudinal disease history, treatment trajectories, performance status, comorbidities, laboratory parameters, radiological findings, molecular biomarkers, and pathology-derived features into a single repository-driven system. Histopathological assessment provides the structural foundation for disease characterization through established prognostic markers such as Breslow thickness, ulceration, and mitotic rate, while molecular profiling identifies oncogenic drivers and therapeutically actionable targets. In parallel, AI-assisted digital pathology enables quantitative analysis of tissue architecture and spatially resolved biological features, while proteomic profiling provides functional information regarding tumor biology, immune activity, and treatment-related adaptations. Radiological imaging modalities, including CT, PET-CT, and MRI, contribute to whole-body assessment of disease burden and progression, complementing laboratory parameters such as LDH and organ function markers that reflect systemic disease status.

The integration of these complementary data layers creates a scalable learning system in which each patient contributes to an expanding knowledge base that continuously refines predictive models and decision-support capabilities. Such multimodal frameworks are increasingly aligned with emerging concepts, including oncological digital twins and AI-assisted multidisciplinary tumor boards, where complex clinical and biological information can be synthesized into actionable recommendations [6,7,30]. Within this context, the Illustrative Institutional Pipeline aims to generate a unified decision-making output that supports evidence-based therapy selection, prediction of therapeutic response, risk stratification, and adaptive treatment planning. Importantly, the framework should be viewed as a translational vision that requires prospective validation before clinical implementation but illustrates how future oncology practice may evolve toward continuously learning, multimodal, and patient-centered decision-support systems.

### 3.3. Implementation of Illustrative Institutional Pipeline in Early-Stage Melanomas

Over the past decade, outcomes for patients with stage I–II melanoma have improved, largely driven by earlier detection, improved staging, and advances in surveillance and adjuvant immunotherapy, particularly for high-risk stage II disease. The largest treatment-driven survival gains are still most visible in stages III–IV, but high-risk stage II is now changing because adjuvant anti–PD-1 therapy, which improves recurrence-free survival and overall survival benefits, is still maturing. Although most patients with stage I–II melanoma experience favorable outcomes [39], a small subset subsequently develops recurrence or metastatic disease despite initially reassuring clinical features. This represents a significant unmet clinical need, as many patients undergo prolonged surveillance despite a relatively low likelihood of progression, while high-risk individuals may remain unidentified. In this context, multimodal approaches integrating AI-assisted digital pathology, spatial proteomics, and clinical data could potentially improve biological risk stratification beyond conventional staging systems. However, several practical challenges remain, including the limited availability of molecular profiling, the absence of routine imaging and multi-omics analyses in most early-stage patients, and the associated costs and infrastructure requirements. Therefore, prospective validation studies are needed to determine whether the improved prognostic precision offered by these technologies justifies their implementation in early-stage disease. Notably, preliminary findings from our melanoma program suggest that AI-assisted histopathology combined with spatial proteomics can identify metabolic and immune-related differences among stage I–II melanomas, supporting the hypothesis that biologically relevant high-risk subgroups can be detectable before clinical progression becomes evident [18]. Such approaches may ultimately enable more personalized surveillance strategies, reduce unnecessary follow-up in low-risk patients, and facilitate earlier intervention in those at greatest risk of recurrence.

### 3.4. Limitations and Future Directions

Importantly, future clinical implementation of multimodal oncology frameworks is expected to evolve as an integrated extension to current diagnostic practice, complementing existing clinical, pathological, and radiological assessment with biologically informed molecular readouts to support more precise patient treatment. In several leading US and European hospitals and precision oncology centers, early implementation of molecular tumor boards integrating genomics, proteomics, digital pathology, and AI-assisted analytical platforms has already begun, supporting the transition toward multimodal clinical decision-making [31,32,40,41,42]. As hospital infrastructures continue to advance, these fields are anticipated to become increasingly integrated into standardized clinical workflows and next-generation tumor board systems. Although current implementation remains limited by requirements for specialized instrumentation, computational infrastructure, data interoperability, and expert bioinformatic support, ongoing technological development is expected to improve scalability, automation, and clinical accessibility. Similarly, advances in explainable AI and harmonized data standards will be essential to strengthen interpretability, clinical trust, and regulatory acceptance. Collectively, these developments support a future transition toward adaptive multimodal diagnostics capable of enhancing patient stratification and treatment selection and are expected to undergo continued scaling and progressive implementation into routine clinical oncology practice.

## 4. Functional Molecular Evidence for Integrated Molecular and Morphological Pathology in Melanoma

### 4.1. The Increasing Role of Proteomics

Although transcriptomic profiling has become an important component of molecular characterization in melanoma and has demonstrated clinical utility for prognostic assessment, it was not included as a primary component of the framework proposed in this manuscript. Nevertheless, the modest correlation between mRNA and protein expression observed across clinical cancer studies (~0.3–0.5) [43] underscores the growing importance of Functional Genomics and functionally validated molecular profiling, increasingly advanced by Pharma, Biotech, regulatory agencies including the FDA, and Academia to better capture biologically actionable disease mechanisms. Protein-based analyses provide a more direct representation of cellular function, reflecting not only gene expression but also post-transcriptional, translational, and post-translational regulatory mechanisms. Furthermore, spatial proteomics enables compartment-specific characterization of tumor, stromal, and immune microenvironments, offering tissue-contextual insights that are generally not accessible through bulk transcriptomic approaches. Importantly, the proposed framework should not be viewed as an alternative to transcriptomic profiling but rather as a complementary strategy, and future integration of gene expression data represents a logical extension of multimodal precision oncology approaches.

### 4.2. Clinicopathology Remains Necessary, but Biologically Incomplete

Conventional clinicopathological variables, including Breslow thickness, mitotic rate, and comorbidity burden, remain the backbone of melanoma assessment but capture only part of the biological landscape driving recurrence and progression [44,45]. Melanoma heterogeneity extends well beyond mutational diversity, encompassing distinct malignant cell states such as MITF-high and AXL-high populations, variable immune, stromal, and endothelial programs, and drug-resistance phenotypes that emerge from interacting cellular ecosystems rather than tumor genotype alone [46,47]. A meaningful fraction of high-risk melanoma therefore remains biologically under-resolved when assessment is restricted to morphology and routine clinical descriptors, supporting the need for deeper molecular readouts integrated with tissue context.

### 4.3. Mitochondrial Rewiring Is a Recurrent Axis of Aggressiveness

**Figure 3:**
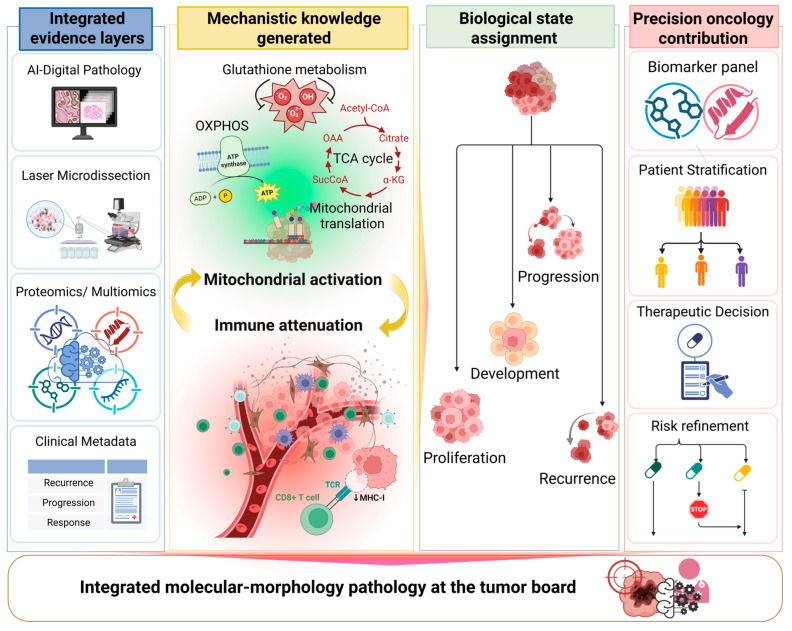
Integrated evidence layers in spatial proteomics provide a unified, high-resolution view of tumor biology by combining morphology, molecular function, and clinical context within the same tissue framework.

Across our studies, mitochondrial rewiring consistently emerges as a defining axis of aggressiveness [18]. In aggressive melanomas, OXPHOS, mitochondrial translation, TCA cycle activation, and RNA polymerase III-linked transcriptional programs are recurrently enriched, particularly in *BRAF V600E*-mutant disease and lesions linked to distant metastasis [48,49]. It should be noted that these mitochondrial observations are well-established in the field. The contribution of our integrated approach lies in their compartment-resolved spatial quantification through AI-based histopathology connected to laser microdissection (LMD). This links mitochondrial rewiring to co-occurring immune attenuation, stromal remodeling, and clinically defined recurrence endpoints, since resolution is not accessible through bulk molecular profiling or morphological assessment alone [18]. In recurrence-prone tumors, this coupled biological state, characterized by mitochondrial and proliferative reinforcement within tumor cells alongside immune-attenuated stromal mircoenvironment, represents precisely the type of biology that conventional pathology cannot resolve. Its identification is directly relevant to tumor board interpretation of disease progression and mechanisms of therapeutic escape (Figure 3). Importantly, these multimodal analytical approaches are already being evaluated in patient cohorts within our respective hospital-based precision oncology studies [5,18].

### 4.4. Intrapatient Heterogeneity Limits Single-Lesion Interpretation

At the protein level, spatially resolved profiling adds mechanistic specificity, linking high-risk disease to mitochondrial translation, PD-1 signaling, and stromal remodeling. Moreover subtype-specific analyses in acral melanoma have identified distinct translational, bioenergetic, and extracellular matrix-remodeling programs involving proteins such as TNC, POSTN, EIF4A1, ARF4, and ATP5IF1 [50]. Furthermore, intra-patient proteogenomic heterogeneity across distant metastases, minimal at the morphological level yet substantial at the functional level, demonstrates that single-lesion sampling underestimates the true extent of disease diversity and that tumor board interpretation should incorporate biological-state information rather than assuming equivalence among histologically similar lesions [51].

### 4.5. Why This Evidence Matters for Integrated Tumor Boards

Taken together, the evidence supports a coherent view of melanoma as a disease organized along interacting mitochondrial, immune, and stromal axes. Clinicopathological variables define part of the risk landscape, but they do not fully capture whether a lesion is mitochondrially reinforced, immune attenuated, stromally remodeled, highly proliferative, or biologically discordant from other lesions in the same patient. Proteogenomics and digital pathology become most useful when they are integrated precisely at that level: not as separate data streams, but as complementary views of tumor state. For tumor boards, this is the clinically relevant message. What is needed is not more data in the abstract, but a formal interpretive layer that can translate morphology, tissue composition, and molecular programs into biologically meaningful state descriptors. In melanoma, the functional evidence now strongly suggests that this integrated layer is not an optional refinement but increasingly necessary for recurrence-risk refinement, resistance framing, and more informed treatment strategies [52].

## 5. Digital Computational Pathology and AI Imaging

### 5.1. Workflow Procedure and the QuPath Pathology Platform

Building on large-scale melanoma studies within the European Cancer Moonshot program [53,54], we have developed a global melanoma imaging atlas integrating high-resolution digital pathology with spatially resolved proteomics to create a publicly accessible resource for next-generation tumor characterization. Our standardized workflow uses QuPath v0.6.0 for whole-slide image analysis, in which each digitized section is divided into 1024 × 1024 tiles and analyzed using a a trained nnU-Net model for pixel-level semantic segmentation of tumor, stroma, necrosis, regression zones, tumor-infiltrating lymphocytes, and adjacent tissue. The model was pretrained on the PUMA (Panoptic segmentation of nUclei and tissue in advanced MelanomA) dataset and fine-tuned on our institutional melanoma cohort through transfer learning [55,56]. All input images are expected to conform to 8-bit depth at a consistent acquisition magnification prior to ingestion. The pipeline automates preprocessing, augmentation, and 5-fold cross-validation with ensemble inference across all folds. Annotations operate at two complementary levels: (1) compartment-level delineation of major tissue regions and (2) cellular-level mapping of tumor nests, stromal populations, and tumor-infiltrating lymphocytes, enabling automated quantification of Breslow thickness, invasion depth, and spatial tissue distribution while reducing inter-observer variability across large cohorts.

The final segmentation results are then re-assembled and displayed within QuPath v0.6.0 as detailed, spatially mapped annotations [57,58,59]. These can be easily reviewed, refined, and validated by expert pathologists, ensuring both efficiency and clinical reliability.

**Figure 4:**
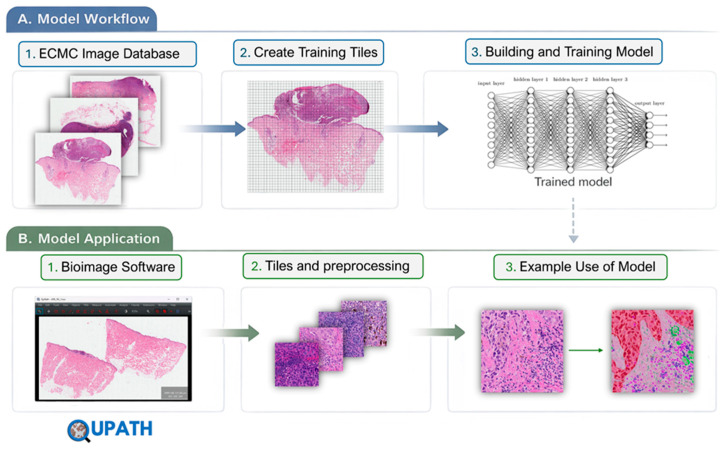
Workflow of digital computational pathology (**A**,**B**): from image acquisition to clinical implementation. This schematic illustrates the end-to-end workflow of digital computational pathology, beginning with high-resolution whole-slide image acquisition from scanned tissue sections and progressing through standardized preprocessing steps, including quality control, stain normalization, and image tiling. AI-driven models are then applied for segmentation, feature extraction, and spatial analysis, generating quantitative representations of tumor architecture, cellular composition, and microenvironmental context. These outputs are integrated with complementary data layers, such as proteomics, molecular profiling, and clinical metadata, within a centralized repository framework. (ECMC: European Cancer Moonshot Center).

The workflow of digital computational pathology, going from image to clinical use, is depicted in Figure 4, where the schematic outline shows the end-to-end pipeline from whole-slide image acquisition through preprocessing (QC, normalization, and tiling) to AI-driven segmentation, feature extraction, and spatial analysis.

**Figure 5:**
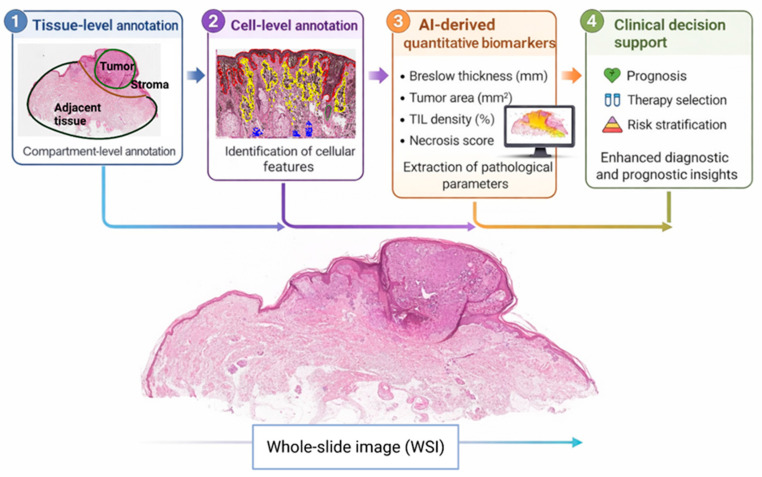
Overview of a 4-step digital computational pathology strategy for predictive treatment guidance, designed for predictive treatment planning and clinical decision-making. Step 1: Tissue-level annotation: whole-slide images are systematically annotated to delineate major compartments, including tumor, stroma, and adjacent tissue, providing a structured map of tumor architecture and microenvironmental context. Step 2: Cellular-level annotation and quantification: high-resolution analysis enables identification and classification of cellular components, including tumor cells, stromal populations, and immune infiltrates. AI-supported models extract quantitative biomarkers reflecting spatial organization, heterogeneity, and functional states. Step 3: Clinical integration and decision support: derived features are integrated with molecular data and clinical metadata to generate actionable insights. This supports Step 4: Prognosis assessment, risk stratification, and therapy selection: forming a predictive roadmap for personalized patient management.

Quantitative outputs on tumor architecture and microenvironment are integrated with molecular data and clinical metadata within a centralized framework. The resulting multimodal insights are translated into structured outputs for routine pathology, supporting diagnosis, prognosis, and tumor board decision-making, while continuous data integration may support iterative model improvement (Figure 5).

### 5.2. Handling Stain Variation in Tumor Images

Stain variation represents a fundamental source of domain shift in computational pathology, arising from differences in reagent composition, staining duration, tissue fixation protocols, and scanner-specific color rendering across institutions and time points. Without explicit handling, these variations introduce systematic bias into model training and reduce generalizability at inference, as the network learns chromatic features that are specific to a particular laboratory or scanner rather than underlying tissue morphology. To address this, we implement RandStainNA [60] as a training-time stain augmentation strategy, in which stain statistics are randomly sampled from a learned distribution during each training iteration rather than normalizing all images to a fixed reference template. This approach encourages the model to develop stain-invariant feature representations, improving robustness to the chromatic heterogeneity inherent in multi-site melanoma datasets. RandStainNA operates in the haematoxylin-eosin-DAB color space and samples augmentation parameters from distributions derived from the training corpus, preserving biologically meaningful morphological variation while decorrelating the model from stain-specific intensity patterns. At inference, a consistent Macenko normalization step is applied to each whole-slide image prior to tiling, using a reference stain matrix derived from a representative subset of our institutional slides, ensuring stable chromatic input conditions without dependence on a fixed external template. This combined strategy, distributional augmentation during training and deterministic normalization at inference, addresses both the training distribution problem and the deployment consistency problem in a principled manner. Taken together, RandStainNA constitutes the primary strategy for handling stain variation within this framework, ensuring that segmentation performance is driven by morphological tissue characteristics rather than laboratory-specific staining artifacts. This approach is particularly well suited to the multi-site composition of the training data, where the chromatic distance between images would otherwise represent a persistent source of domain shift that fixed-reference normalization methods are ill-equipped to handle consistently.

## 6. Concluding Remarks

In conclusion, the increasing transition from primary melanoma to recurrent metastatic disease highlights a fundamental limitation in current clinical practice: the absence of an integrated, longitudinal, and functionally informed understanding of tumor evolution. From our clinical centers, we see that patient survival improves through immunotherapy and targeted treatments; melanoma is increasingly managed as a dynamic and long-term disease, necessitating more advanced, adaptive, and biologically grounded decision-support frameworks. The proposed multimodal disease profiling (MDP) approach can directly address this need by bridging molecular science with clinical application and could support decision-making.

By integrating proteogenomics, AI-driven digital pathology, and structured longitudinal clinical metadata, MDP could facilitate a comprehensive and continuously evolving representation of each patient’s disease. This framework moves beyond static diagnostic snapshots toward a systems-level understanding of tumor behavior, capturing signaling dynamics, spatial heterogeneity, immune interactions, and microenvironmental adaptations. Importantly, it allows the identification of functional tumor states that underlie progression, therapeutic response, and resistance, features that are not sufficiently resolved through conventional pathology or genomics alone.

Our work, supported by large-scale melanoma cohorts and infrastructure within the European Cancer Moonshot Center, demonstrates that clinically relevant tumor behavior is governed by coordinated biological programs, including mitochondrial activation, immune modulation, and stromal remodeling. These interconnected axes define disease aggressiveness more accurately than single biomarkers or morphological features. The integration of spatial histopathology with proteome-level data can further support the distinction between apparent therapeutic response and latent metastatic potential, addressing a critical unmet need in risk stratification and treatment planning. The development of a global melanoma imaging atlas and standardized AI-driven pathology workflows provides a scalable and reproducible foundation for implementing MDP in clinical settings. This resource, integrated with high-quality multimodal data, enables the discovery of actionable biological patterns and the AI-assisted prediction of disease trajectories. Its true value, however lies not in generating of ever-increasing volumes of data, but in establishing of an interpretive framework that transforms complex biological and clinical information into clinically meaningful knowledge. For multidisciplinary tumor boards, this marks a fundamental shift, from fragmented, discipline-specific data interpretation to integrated, biologically, informed decision-making. Rather than representing a simple technological refinement this approach reflects a necessary evolution in precision oncology, with the potential to meaningfully enhance patient outcomes, preserve quality of life, and extend long-term survival for cancer patients.

## Figures and Tables

**Table 1:** Trends for consolidated melanoma-specific outcomes.

Metric—Melanoma	Changes	2015 → 2025 Trend
Metastatic incidence	↑	~25%
Stage IV at diagnosis	↑	~4%
Recurrence → metastasis	-	Stable biologically
Patients living with metastasis	↑	Strong (≈2–3×)
Survival in metastatic disease	↑	Major improvement (2×)

## Data Availability

Raw proteomics data from previously published studies within this program are publicly available through the repositories specified in the original publications, in full compliance with the Swedish Biobank Law and BBMRI guidelines [5,18,44,45,48,49,50]. Additional data supporting the findings of this perspective are available from the corresponding author upon reasonable request. Code availability: The QuPath v0.6.0 scripting workflows and nnU-Net configuration files used for the AI-based segmentation pipeline described in Section 5 are available upon request from the corresponding author and will be deposited in a public repository upon completion of ongoing validation studies. Model availability: The pretrained segmentation model, fine-tuned on our institutional melanoma dataset, will be made publicly available upon completion of validation.

## References

[1] Arnold M., Singh D., Laversanne M., Vignat J., Vaccarella S., Meheus F., Cust A.E., De Vries E., Whiteman D.C., Bray F. (2022). Global Burden of Cutaneous Melanoma in 2020 and Projections to 2040. JAMA Dermatol..

[2] Wang M., Gao X., Zhang L. (2025). Recent Global Patterns in Skin Cancer Incidence, Mortality, and Prevalence. Chin. Med. J..

[3] (2026). American Cancer Society—Key Statistics for Melanoma Skin Cancer. https://www.cancer.org/cancer/types/melanoma-skin-cancer/about/key-statistics.html.

[4] (2025). AOC25 Why Northern Europe Has the Highest Skin Cancer Rates. https://www.aoc25.com/post/why-northern-europe-has-the-highest-skin-cancer-rates.

[5] Fülöp L., Szigeti B., Guedes J., Woldmar N., Oskolás H., Marko-Varga M., Appelqvist R., Wieslander E., Pawlowski K., Szadai L. (2026). Proteomics-Enhanced AI-Digital Pathology in Metastatic Mucinous Colorectal Carcinoma: A Case Report. bioRxiv.

[6] Nardone V., Marmorino F., Germani M.M., Cichowska-Cwalińska N., Menditti V.S., Gallo P., Studiale V., Taravella A., Landi M., Reginelli A. (2024). The Role of Artificial Intelligence on Tumor Boards: Perspectives from Surgeons, Medical Oncologists and Radiation Oncologists. Curr. Oncol..

[7] Wang X., Wang Q., Ding G., Wang J., Tang Y., Feng Y. (2025). Artificial Intelligence in Multidisciplinary Tumor Boards Enhancing Decision Making and Clinical Outcomes in Oncology. iScience.

[8] (2026). EORTC—Quality of Life—Questionnaires. https://qol.eortc.org/questionnaires/.

[9] Mani D.R., Krug K., Zhang B., Satpathy S., Clauser K.R., Ding L., Ellis M., Gillette M.A., Carr S.A. (2022). Cancer Proteogenomics: Current Impact and Future Prospects. Nat. Rev. Cancer.

[10] Khan S.N., Danishuddin, Khan M.W.A., Guarnera L., Akhtar S.M.F. (2026). Multi-Modal AI in Precision Medicine: Integrating Genomics, Imaging, and EHR Data for Clinical Insights. Front. Artif. Intell..

[11] Park K.C., Yoo W. (2026). Translating Multimodal Foundation Models into Oncology: Toward a Future Where AI Directs Diagnosis and Therapy. Genes Dis..

[12] Venturi F., Veronesi G., Gualandi A., Magnaterra E., Scotti B., Sotiri I., Baraldi C., Alessandrini A.M., Veneziano L., Vaccari S. (2025). From Slide to Insight: The Emerging Alliance of Digital Pathology and AI in Melanoma Diagnostics. Cancers.

[13] Waqas A., Tripathi A., Ramachandran R.P., Stewart P.A., Rasool G. (2024). Multimodal Data Integration for Oncology in the Era of Deep Neural Networks: A Review. Front. Artif. Intell..

[14] Hodi F.S., Chiarion-Sileni V., Gonzalez R., Grob J.-J., Rutkowski P., Cowey C.L., Lao C.D., Schadendorf D., Wagstaff J., Dummer R. (2018). Nivolumab plus Ipilimumab or Nivolumab Alone versus Ipilimumab Alone in Advanced Melanoma (CheckMate 067): 4-Year Outcomes of a Multicentre, Randomised, Phase 3 Trial. Lancet Oncol..

[15] (2025). Melanoma Updates—Epidemiology, Diagnostics, and Treatment. https://practicaldermatology.com/issues/may-june-2025/melanoma-updates-epidemiology-diagnostics-and-treatment/35917/?utm_source=chatgpt.com.

[16] Van Not O.J., Van Den Eertwegh A.J.M., Jalving H., Bloem M., Haanen J.B., Van Rijn R.S., Aarts M.J.B., Van Den Berkmortel F.W.P.J., Blank C.U., Boers-Sonderen M.J. (2024). Long-Term Survival in Patients With Advanced Melanoma. JAMA Netw. Open.

[17] Noringriis I.M., Donia M., Madsen K., Schmidt H., Haslund C.A., Bastholt L., Svane I.M., Ellebaek E. (2025). Long-Term Clinical Outcome of Patients with Metastatic Melanoma and Initial Stable Disease during Anti-PD-1 Checkpoint Inhibitor Immunotherapy with Pembrolizumab. Br. J. Cancer.

[18] Szadai L., Guedes J.D.S., Woldmar N., De Almeida N.P., Jánosi Á.J., Rajeh A., Kovács F., Kriston A., Migh E., Wan G. (2023). Mitochondrial and Immune Response Dysregulation in Melanoma Recurrence. Clin. Transl. Med..

[19] Lipkova J., Chen R.J., Chen B., Lu M.Y., Barbieri M., Shao D., Vaidya A.J., Chen C., Zhuang L., Williamson D.F.K. (2022). Artificial Intelligence for Multimodal Data Integration in Oncology. Cancer Cell.

[20] (2025). BioRender. https://www.biorender.com.

[21] (2026). European Cancer Organisation. https://www.europeancancer.org/.

[22] (2026). National Comprehensive Cancer Network. https://www.nccn.org/.

[23] Everhov Å.H., Frisell T., Osooli M., Brooke H.L., Carlsen H.K., Modig K., Mårild K., Lindström J., Sköldin K., Heurgren M. (2025). Diagnostic Accuracy in the Swedish National Patient Register: A Review Including Diagnoses in the Outpatient Register. Eur. J. Epidemiol..

[24] Murphy P.K., Sellers M.E., Bonds S.H., Scott S. (2024). The SEER Program’s Longstanding Commitment to Making Cancer Resources Available. JNCI Monogr..

[25] Caffrey M. (2021). Flatiron Health Leverages Expertise with Real-World Data to Examine Cancer Care Disparities. Am. J. Manag. Care.

[26] Raab R., Küderle A., Zakreuskaya A., Stern A.D., Klucken J., Kaissis G., Rueckert D., Boll S., Eils R., Wagener H. (2023). Federated Electronic Health Records for the European Health Data Space. Lancet Digit. Health.

[27] Wan G., Nguyen N., Liu F., DeSimone M.S., Leung B.W., Rajeh A., Collier M.R., Choi M.S., Amadife M., Tang K. (2022). Prediction of Early-Stage Melanoma Recurrence Using Clinical and Histopathologic Features. npj Precis. Oncol..

[28] Wan G., Leung B.W., DeSimone M.S., Nguyen N., Rajeh A., Collier M.R., Rashdan H., Roster K., Zhou X., Moseley C.B. (2024). Development and Validation of Time-to-Event Models to Predict Metastatic Recurrence of Localized Cutaneous Melanoma. J. Am. Acad. Dermatol..

[29] Andrew T.W., Combalia M., Hernandez C., Grant S., Paragh G., Puig S., Mc Arthur G., Richardson G., Sloan P., Shalhout S.Z. (2025). Multimodal AI and Tumour Microenvironment Integration Predicts Metastasis in Cutaneous Melanoma. Nat. Commun..

[30] Liu F., Beck S., Yang L., Luo H., Zhang K. (2026). Advancing AI for Multi-Omics and Clinical Data Integration in Basic and Translational Cancer Research. Nat. Rev. Cancer.

[31] Tamborero D., Dienstmann R., Rachid M.H., Boekel J., Lopez-Fernandez A., Jonsson M., Razzak A., Braña I., De Petris L., Yachnin J. (2022). The Molecular Tumor Board Portal Supports Clinical Decisions and Automated Reporting for Precision Oncology. Nat. Cancer.

[32] (2024). Cedars Sinai Crafting Technologies to Reshape the Future of Medicine. https://pulse.cedars-sinai.org/news/crafting-technologies-to-reshape-the-future-of-medicine.

[33] (2026). Cedars Sinai Biomarkers for Precision Oncology. https://www.cedars-sinai.edu/health-sciences-university/research/departments-institutes/translational-research/biomarkers-for-precision-oncology.html.

[34] Keller R.B., Mazor T., Sholl L., Aguirre A.J., Singh H., Sethi N., Bass A., Nagaraja A.K., Brais L.K., Hill E. (2023). Programmatic Precision Oncology Decision Support for Patients With Gastrointestinal Cancer. JCO Precis. Oncol..

[35] (2026). The LOOP Zurich Medical Research Center. https://theloopzurich.ch/en/focal-points/incubator-projects/ai-tumor-board-personalized-decision-support-in-precision-oncology/.

[36] Wang X., Zhao J., Marostica E., Yuan W., Jin J., Zhang J., Li R., Tang H., Wang K., Li Y. (2024). A Pathology Foundation Model for Cancer Diagnosis and Prognosis Prediction. Nature.

[37] Pao J.J., Biggs M., Duncan D., Lin D.I., Davis R., Huang R.S.P., Ferguson D., Janovitz T., Hiemenz M.C., Eddy N.R. (2023). Predicting EGFR Mutational Status from Pathology Images Using a Real-World Dataset. Sci. Rep..

[38] Bossard C., Salhi Y., Chetritt J., Salhi S. (2024). 1121P Artificial Intelligence to predict BRAF mutational status from whole slide images in melanoma. Ann. Oncol..

[39] Garbe C., Keim U., Amaral T., Berking C., Eigentler T.K., Flatz L., Gesierich A., Leiter U., Stadler R., Sunderkötter C. (2022). Prognosis of Patients With Primary Melanoma Stage I and II According to American Joint Committee on Cancer Version 8 Validated in Two Independent Cohorts: Implications for Adjuvant Treatment. J. Clin. Oncol..

[40] Moore J.H., Li X., Chang J.H., Tatonetti N.P., Theodorescu D., Chen Y., Asselbergs F.W., Venkatesan M., Wang Z.P. (2024). SynTwin: A Graph-Based Approach for Predicting Clinical Outcomes Using Digital Twins Derived From Synthetic Patients. Pac. Symp. Biocomput. Pac. Symp. Biocomput..

[41] Nguyen C., Sharif-Afshar A.-R., Fan Z., Xie Y., Wilson S., Bi X., Payor L., Saouaf R., Kim H., Li D. (2016). 3D High-Resolution Diffusion-Weighted MRI at 3T: Preliminary Application in Prostate Cancer Patients Undergoing Active Surveillance Protocol for Low-Risk Prostate Cancer. Magn. Reson. Med..

[42] Qureshi T.A., Gaddam S., Wachsman A.M., Wang L., Azab L., Asadpour V., Chen W., Xie Y., Wu B., Pandol S.J. (2022). Predicting Pancreatic Ductal Adenocarcinoma Using Artificial Intelligence Analysis of Pre-Diagnostic Computed Tomography Images. Cancer Biomark. Sect. Dis. Markers.

[43] Ponomarenko E.A., Krasnov G.S., Kiseleva O.I., Kryukova P.A., Arzumanian V.A., Dolgalev G.V., Ilgisonis E.V., Lisitsa A.V., Poverennaya E.V. (2023). Workability of mRNA Sequencing for Predicting Protein Abundance. Genes.

[44] Guedes J., Szadai L., Woldmar N., Jánosi Á.J., Koroncziová K., Lengyel B.M., Kelemen B., Boltas E., Gyulai R., Wieslander E. (2025). The Melanoma MEGA-Study: Integrating Proteogenomics, Digital Pathology, and AI-Analytics for Precision Oncology. J. Proteom..

[45] Guedes J., Woldmar N., Szasz A.M., Wieslander E., Pawłowski K., Horvatovich P., Malm J., Szadai L., Németh I.B., Marko-Varga G. (2025). A Perspective on Integrating Digital Pathology, Proteomics, Clinical Data and AI Analytics in Cancer Research. J. Proteom..

[46] Grzywa T.M., Paskal W., Włodarski P.K. (2017). Intratumor and Intertumor Heterogeneity in Melanoma. Transl. Oncol..

[47] Tirosh I., Izar B., Prakadan S.M., Wadsworth M.H., Treacy D., Trombetta J.J., Rotem A., Rodman C., Lian C., Murphy G. (2016). Dissecting the Multicellular Ecosystem of Metastatic Melanoma by Single-Cell RNA-Seq. Science.

[48] Kuras M., Betancourt L.H., Hong R., Szadai L., Murillo J.R., Horvatovich P., Parada I.P., Eriksson J., Szeitz B., Deszz B. (2023). Histopathology-Assisted Proteogenomics Provides Foundations for Stratification of Melanoma Metastases. bioRxiv.

[49] de Almeida N.P., Jánosi Á.J., Hong R., Rajeh A., Nogueira F., Szadai L., Szeitz B., Parada I.P., Doma V., Woldmar N. (2024). Mitochondrial Dysfunction and Immune Suppression in BRAF V600E-Mutated Metastatic Melanoma. Clin. Transl. Med..

[50] Horvatovich P., Fernandez-Coto D.L., Ayala M., Alonso R., Palmqvist A., Oskolas H., Christersson L., Fulöp L., Szigeti B., Marko-Varga M. (2026). AI-Accelerated Acoustic FFPE Proteomics Identifies ATP5IF1-Linked Mitochondrial Remodeling as a Defining Feature of Acral Melanoma.

[51] Szeitz B., Hagemeijer Y.P., Pahi Z.G., Ujfaludi Z., Kuras M., Rodriguez J., Doma V., Mohacsi R., Herold M., Herold Z. (2025). Distant Metastases of Melanoma Exhibit Varying Extent of Intrapatient Proteogenomic Heterogeneity. Clin. Transl. Med..

[52] Harel M., Ortenberg R., Varanasi S.K., Mangalhara K.C., Mardamshina M., Markovits E., Baruch E.N., Tripple V., Arama-Chayoth M., Greenberg E. (2019). Proteomics of Melanoma Response to Immunotherapy Reveals Mitochondrial Dependence. Cell.

[53] (2026). Cancer Moonshot Lund Center. http://www.cancermoonshotlund.com/index.php/2-home-cancer-moonshot-us/.

[54] (2026). Cancer Moonshot Initiative. https://www.cancer.gov/research/progress/moonshot-cancer-initiative.

[55] Bankhead P., Loughrey M.B., Fernández J.A., Dombrowski Y., McArt D.G., Dunne P.D., McQuaid S., Gray R.T., Murray L.J., Coleman H.G. (2017). QuPath: Open Source Software for Digital Pathology Image Analysis. Sci. Rep..

[56] Schuiveling M., Liu H., Eek D., Breimer G.E., Suijkerbuijk K.P.M., Blokx W.A.M., Veta M. (2025). A Novel Dataset for Nuclei and Tissue Segmentation in Melanoma with Baseline Nuclei Segmentation and Tissue Segmentation Benchmarks. GigaScience.

[57] Sauter D., Lodde G., Nensa F., Schadendorf D., Livingstone E., Kukuk M. (2023). Deep Learning in Computational Dermatopathology of Melanoma: A Technical Systematic Literature Review. Comput. Biol. Med..

[58] Acs B., Rantalainen M., Hartman J. (2020). Artificial Intelligence as the next Step towards Precision Pathology. J. Intern. Med..

[59] Comes M.C., Fucci L., Mele F., Bove S., Cristofaro C., De Risi I., Fanizzi A., Milella M., Strippoli S., Zito A. (2022). A Deep Learning Model Based on Whole Slide Images to Predict Disease-Free Survival in Cutaneous Melanoma Patients. Sci. Rep..

[60] Wang C., Li S., Ke J., Zhang C., Shen Y. (2024). RandStainNA++: Enhance Random Stain Augmentation and Normalization Through Foreground and Background Differentiation. IEEE J. Biomed. Health Inform..

[61] ICPC. https://icpc.cancer.gov/portal/.

[62] OpenAI ChatGPT (GPT-5.1) [Large Language Model]. https://chatgpt.com.

